# Study on the binding behavior and functional properties of soybean protein isolate and β-carotene

**DOI:** 10.3389/fnut.2022.984490

**Published:** 2022-09-08

**Authors:** Yating Zhang, Wenqi Zhao, Zhuqing Xing, Beibei Zhu, Ruiyang Hou, Junxi Zhang, Taoran Li, Zifan Zhang, Hongwu Wang, Zheng Li

**Affiliations:** ^1^College of Healthy Science and Engineering, Tianjin University of Traditional Chinese Medicine, Tianjin, China; ^2^College of Chinese Medicine Pharmaceutical Engineering, Tianjin University of Traditional Chinese Medicine, Tianjin, China

**Keywords:** soybean protein isolate, β-carotene, multi-spectroscopic techniques, molecular docking, emulsifying properties

## Abstract

This study focused on the non-covalent interaction between soybean protein isolate (SPI) and β-carotene (BC). The conformational changes of SPI with β-carotene in varying proportions (BC/SPI: 2%, 4%, 6%, 8%, and 10%) were investigated by multi-spectroscopy and molecular docking. Results showed that the quenching mode is static quenching and binding affinity increased with temperature. The stoichiometry was 1:1, indicating there was only one binding site in SPI. The binding was based on entropy and primarily driven by hydrophobic interactions and its binding constant was in the order of 10^4^ L⋅mol^–1^. The addition of the β-carotene affected the secondary structure of SPI resulting in an increase in α-Helix and a decrease in random coil and β-turn content, indicating protein aggregated and hydrophobic interactions occurred. Sodium dodecyl sulfate-polyacrylamide gel electrophoresis (SDS-PAGE) verified that no new larger molecular weight substance was formed and no covalent interaction existed. Molecular docking corroborated that electrostatic and hydrophobic interactions were both involved in the formation of complexes, where hydrophobic interaction was the dominant one. Moreover, β-carotene improved 1,1-diphenyl-2-picrylhydrazyl (DPPH) radical scavenging activity, foaming capacity, and emulsifying stability of SPI. These findings provide useful information about the interaction mechanism of SPI and β-carotene, which contributes to the further development and application of SPI products rich in β-carotene in the food industry.

## Introduction

β-carotene (C_40_H_56_; BC), a precursor of vitamin A, is a carotenoid widely found in fruits and vegetables. It has plenty of beneficial effects on human health, such as anti-cancer, antioxidants, prevention of cardiovascular disease, and age-related macular degeneration. Therefore, β-carotene is a recognized superior functional food additive (1). However, β-carotene is almost insoluble in water and even hardly dissolved in organic solvents, such as acetone, anhydrous ethanol, dimethyl sulfoxide, and some oils and fats. Additionally, it is easily crystallized at room temperature, and sensitivity to light, heat, and oxygen greatly limits its application in food (2).

Recently, the interaction between β-carotene and proteins, which was conducted to modify the properties of β-carotene to expand its application, has attracted raising interest from researchers. Li et al. (3) studied the interaction of β-carotene and astaxanthin with human serum albumin (HSA) and bovine serum albumins (BSA) under physiological conditions by using multi-spectroscopic techniques. The moderate affinity of the carotenoids and HSA/BSA helps these two carotenoids diffuse from the circulatory system to their target sites. A study by Andresa et al. (4) showed that β-carotene entrapped within protein-coated MCT droplets was more stable than within T80-MCT systems. β-carotene interacted with proteins in emulsions changing their properties and behavior under the gastrointestinal tract, consequently, enhancing the stability/bioaccessibility of β-carotene. Allahdad et al. (5–7) explored the interaction mechanism between β-carotene and whey proteins and caseins to improve the color and taste of milk and the consequences were checked by introducing β-carotene into a hydrated polar solution containing milk protein. They found that although WPI was very effective in protecting β-carotene against photodecomposition *via* insertion into the β-lactoglobulin cavity, the open structure and greater amounts of antioxidative amino acids made casein a better protectant than WPI. The association of β-carotene with milk proteins led to the decrease in electron transfer as opposed to hydrogen transfer to the radical species. To our knowledge, most of the current studies in this field are on animal proteins, and there are few systematic studies on the binding of plant proteins to small molecule active components.

Soybean protein isolate (SPI) is an excellent and cheap plant protein. The varieties and contents of eight amino acids in SPI meet the needs of the human body, which effectively supplemented the essential amino acids (8). SPI is also widely used in the delivery of functional nutrients in food because of its good physicochemical features and nutritional value (5, 9–11). The peptide chains of SPI are rich in hydrophobic amino acid residues and charged amino acid residues, which can bind to functional nutrients through hydrophobic, electrostatic, and hydrogen bonding interactions. SPI can promptly deliver and transport the hydrophobic active substances to the specified position *in vivo*, thus improving the bioavailability of nutrients and enhancing some functional properties of proteins (12). Nevertheless, when different active ingredients are transported, there are great differences in embedding efficiency, stability, and bioavailability. It was found that the binding mode of active components and protein depended on the type and concentration of proteins, the structure of active components as well as their molecular weight, and so on (13, 14). Covalent and non-covalent interactions are two main interaction ways between proteins and nutrients. According to the existing research of Zhang et al., the non-covalent interactions of SPI are more fundamental in extrusion cooking (15), and Y. Chen et al. further explored the effects of the non-covalent interactions between polyphenols and proteins on the formations of the heterocyclic amines in dry heated soybean protein isolate (16). Moreover, Wang et al. (17) researched the physicochemical properties and emulsifying properties during the formation of soybean protein isolate-hawthorn flavonoids non-covalent complexes to provide a theoretical basis for protein processing and application. However, few systematic studies are probing into the interaction mechanism of soy isolate protein and β-carotene.

Based on these findings, the main objective of this work was to comprehensively study the potential binding behavior between SPI and β-carotene and the effects on their functional properties. Accordingly, multi-spectroscopic techniques, including fluorescence spectroscopy, Fourier transform infrared spectroscopy (FTIR), and circular dichroism (CD) spectroscopy, were used to investigate the non-covalent interaction forces, binding site, and the conformation changing patterns of SPI in the presence of β-carotene. Molecular docking was employed to further reveal the mechanism at the molecular level and verify the experimental results. Meanwhile, changes in various functional properties of SPI after binding with β-carotene were detected. It was expected to get further insights into the effect of functional nutrients on the structural and functional properties of SPI, thus, offering a reference for the development of novel protein additives with good functional properties and improved health benefits in the future.

## Materials and methods

### Materials

The β-carotene (99.8 %) and DPPH were purchased from Macklin Biochemical Company (Shanghai, China). SurePAGE, Bis-Tris (4–12%, 12 wells, 10/pk), Tris-MOPS-SDS Running Buffer Powder, and PAGE-MASTER Protein Standard Plus were bought from GenScript (860 Centennial Ave., Piscataway, NJ, United States). The Coomassie brilliant blue fast staining solution and SDS-PAGE loading buffer (with DDT) were achieved by Beijing Solarbio Science and Technology Co., Ltd., (Beijing, China). Potassium bromide was purchased from Comere Chemical Reagents Co., Ltd., (Tianjin, China) and it is in the spectrum of purity. All the reagents above were of analytical grade except otherwise explained.

### Preparation of soybean protein isolate

SPI was extracted according to the method of Y. Zhang (18) and the obtained protein solution was freeze-dried and crushed to yield SPI powder.

### The preparation of complexes

Complexes preparation was in accordance with the method of Deng et al. (19), Teng et al. (20), and Zhang et al. (21), and slightly adjusted. The SPI aqueous solution (10^–5^ M) was fully mixed with β-carotene ethanol solution (0.15 mg/ml) in different weight ratios (SPI/BC: 2%, 4%, 6%, 8%, and 10%). Subsequently, the mixed solution was put in a water bath at 273, 298, 318, and 338 K for 30 min, respectively. After insulation, the complex samples were evaporated by a vacuum rotary evaporator (Sheng-ye, RE-52AA, China) until ethanol was completely removed. Afterward, ultrapure water was added to adjust the volume to the original volume. The experiment was carried out under dark conditions during the whole course.

### Fluorescence spectroscopy

To explore the interaction mechanisms between SPI and β-carotene, a JASCO FP-7100 spectrofluorometer (Jasco, FP-7100, Japan) was used to record the emission spectra at 300–500 nm with the excitation wavelength at 280 nm, respectively. The excitation and emission slits were set to 20 and 5 nm, and the scan speed was 2,400 nm/min. The SPI–β-carotene solutions were pre-warmed at 273, 298, 318, and 338 K for 30 min.

To further explore the fluorescence quenching mechanism, Stern-Volmer (Equation 1) was applied to fit the fluorescence intensity in *λ_*max*_* at different temperatures.


(1) $$\frac{F_{0}}{F}=1+K_{S\,V}\,\left[\beta \right]=1+k_{q}\cdot \sigma_{0}\,\left[Q\right]$$

Here, *F*_0_ and *F* are fluorescence intensities in the absence and presence of β-carotene, respectively. [β] is the concentration of quencher, *k*_*q*_ is the biomolecular quenching constant, σ_0_ is the average biomolecular fluorescence lifetime without quenchers equaling to 10^–8^ s, and *K*_*SV*_ is the Stern-Volmer quenching constant.

A double logarithmic Stern-Volmer equation (Equation 2) was used for detecting the static quenching principle in a liner range at different temperatures.


(2) $$L\,g\,\frac{F_{0}-F}{F}=L\,g\,K_{a}+n\cdot L\,g\,\left[Q\right]$$

Here *F*_0_ and *F* are fluorescence intensities in the absence and presence of β-carotene, respectively. [*Q*] is the concentration of quencher, *n* is the bonding site number of the complex, and *K*_*a*_ is the apparent binding constant.

To find out the thermodynamic parameters of the complex, the Vant-Hoff equation (Equations 3, 4) were put to use to make the liner fit.


(3) $$l\,n\,K_{a}=-\frac{△\,H^{0}}{R\,T}+\frac{△\,S^{0}}{R}$$

(4) $$△\,G^{0}=△\,H^{0}-T\cdot △\,S^{0}$$

Here *K*_*a*_ is the apparent binding constant. △*H*^0^, △*S*^0^, and △*G*^0^ respond to enthalpy change, entropy change, and Gibbs free energy change, successively. *R* is gas constant (*R* = 8.314J⋅*mol*^−1^⋅*K*^−1^), and *T* is standard thermodynamic temperature.

### Fourier transform infrared spectroscopy

The infrared spectrum of SPI, BC, and SPI–BC complex was measured by a spectrum instrument (Bruker, Germany). Briefly, the sample powder (1 mg) was mixed with KBr powder (150 mg), ground, and pressed into a transparent flake. A total of 32 scans were taken at 4 cm^–1^ resolution from 4,000 to 400 cm^–1^. Background interference was eliminated *via* KBr and the work environment was 25°C (22, 23).

### Circular dichroism spectroscopy

The method of Chamani et al. (24, 25) with slight retouch was taken to measure the CD spectroscopy of the liquid samples, which was heat preservation at 25°C by JASCO (J-810, Japan). The spectropolarimeter was set in the region from 250 nm to 165 nm at 25°C. The deltax was −0.05 and the *N* point was 1,701. The concentration was 6 10^–6^ M for all proteins, the mean residue weight was 115 g/mol and a cuvette of 1 mm path length was used. The obtained data were converted to molar ellipticity [θ] (deg⋅cm^2^ ⋅dmol^–1^), according to Equation 5.


(5) $$\left[\theta \right]=\frac{\theta }{R\times C\times l\times 10}$$

Where θ is the CD signal in degree at each wavelength, *C* is protein concentration in mol/L, *l* is the length of light path (cm), and *R* is the residue number of SPI.

The final statistics were analyzed by the CDpro software, and the result is shown in Table 2.

### Sodium dodecyl sulfate polyacrylamide gel electrophoresis

The sodium dodecyl sulfate polyacrylamide gel electrophoresis (SDS-PAGE) of SPI and SPI–BC complex were carried out according to the method of Yan et al. (26). The freeze-dried powder of samples was fully dissolved in 0.5 ml loading buffer to obtain a 100 μM SPI solution and then boiled it for 5 min to unfold the SPI. The sample solution (10 μL) after centrifugation was added to each loading slot successively, and the voltage of laminated gel and separation gel was set as 80 mV and 120 mV, respectively. Coomassie brilliant blue fast staining solutions A and B (v/v = 5:1) were mixed to attain the working solution. The gel was placed into 50 ml boiling deionized water for 5 min and then transferred to a 25 ml boiling working solution for 60 s. Afterward, it was transferred to a decolorizing shaker for 10 min. Finally, the image was obtained by a gel analysis imaging system to analyze molecular weight changes of SPI.

### Modular docking simulation

The modular docking was performed according to the method of Nada et al. (27) with a little alteration. The Glide module of Schrödinger (2021) was applied for molecular docking. The 3D structure of β-carotene (Substance SID: 319295400) was obtained from PubChem^1^ and then optimized by Chem3D 20.0 *via* minimization of energy. The crystal structure of 7S protein (PDB ID:3AUP) and 11S protein (PDB ID:1FXZ) were acquired from the RCSB Protein Data Bank database^2^ and the Protein Preparation Wizard of Maestro 12.7 was conducted to prepare them (28).

The Bind Site Detect and Receptor Grid Generation module was conducted to detect the active binding sites and define the active site grid of target proteins, respectively. After using Ligpre to obtain the Lig Files, ran Ligand and Dock module. All parameters were by default. The final binding conformations were recorded based on the calculated GlideScore and the results were visualized using PyMOL (29).

### Determination of DPPH radical scavenging activity

The DPPH radical scavenging activity of the protein solution with or without BC was determined according to the methodology formulated by Yamamoto (30) with slight modification. Different ratios of BC were added to the samples at 25°C, as mentioned in section “The preparation of complexes.” First, a 2 ml sample solution was mixed with ethanolic DPPH solution at the same proportion. Moreover, a control group mixed with 2 ml ethanol with sample solution at equal volume was set to exclude the impact of the color of BC at the absorbance value. The mixture was vortexed for 1 min and shaken for 30 min in darkness. A double beam UV–visible spectrophotometer (Mapada, Shanghai) was used to achieve the absorbance at 517 nm. The DPPH radical scavenging activity was reckoned with Equation 6.


(6) $$Radicalscavengingactivity\left(\%\right)=\left(1-\frac{A_{C}}{A_{0}}\right)\times 100\%$$

Here, *A*_*C*_ is the absorbance value of the solution with DPPH and *A*_0_ is the value of the blank.

### Foaming capacity and foaming stability

The foaming capacity (FC) and foaming stability (FS) were investigated following the means of Yan (31). The samples (50 ml) were agitated by a high-shear mixer at 16,000 rpm for 40 s to produce the foam and the foaming volume was recorded immediately. The foaming capacity (FC) was calculated by Equation 7:


(7) $$F\,C=\frac{V_{a}-V_{b}}{V_{a}}\times 100$$

where *V*_*a*_ and *V*_*b*_ represent the volumes of the samples with and without whipping, respectively.

Keep the foaming samples standing at room temperature for 20 min and record their volume. Equation 8 was used to calculate the foaming stability (FS).


(8) $$F\,S=\frac{V_{F\,\left(0\right)}-V_{F\,\left(t\right)}}{V_{F\,\left(0\right)}}\times 100$$

Here *V*_*F*(0)_ and *V*_*F*(*t*)_ represent the volumes of the samples standing for 0 min and *t* min after whipping, respectively.

### Emulsifying activity index and emulsifying stability index

The emulsifying activity index (EAI) and emulsifying stability index (ESI) were referred to the technique of Xi et al. (32) with little modification. The sample solution (30 ml) was mixed with 10 ml of soybean oil. Afterward, the mixture was homogenized for 40 s to acquire aimed emulsion. The others operation followed the referenced method. EAI and ESI were calculated by Equations 9, 10.


(9) $$E\,A\,I\,\left(m^{2}/g\right)=\frac{2\times 2.303}{C\times \left(1-\varphi \right)\times 10}$$

(10) $$ESI\left(\%\right)=\frac{A_{10}}{A_{0}}\times 100$$

Here, *C* is the protein concentration (mg/mL) in samples, φ is on behalf of the oil phase fraction (v/v) in emulsion, and 100 represents dilution folds.

### Statistical analysis

All measurements were conducted three times. One-way ANOVA was used for data analysis by the SPSS 21.0.0.0 software, *p* < 0.05 was considered statistically significant. And the image description was completed by the Origin 9.8.2 software.

## Results and discussion

### Fluorescence quenching

**FIGURE 1:**
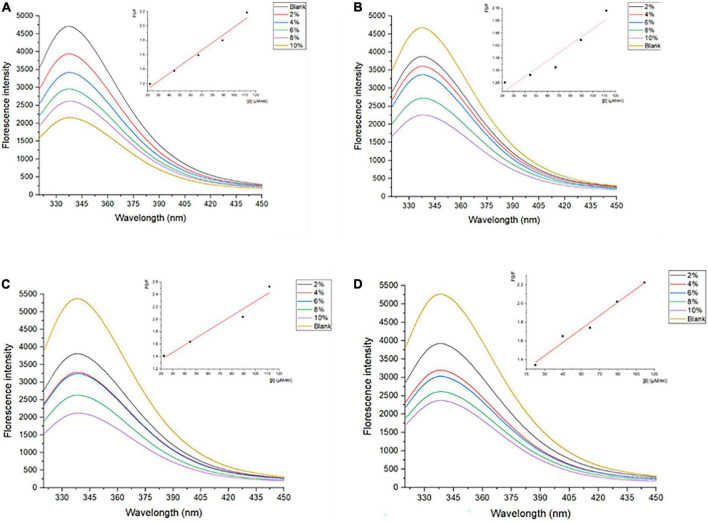
Fluorescence spectra of SPI and SPI–BC complexes at different weight ratios (BC/SPI: 2%, 4%, 6%, 8%, and 10%) at **(A)** 273 K, **(B)** 298 K, **(C)** 318 K, and **(D)** 338 K.

Generally, tryptophan, tyrosine, and phenylalanine residues in protein scan fluoresce to some extent critically dependent on the folding degree of proteins, especially the tryptophan residues (33). The intrinsic fluorescence spectrum reflects the polarity of the microenvironment in which tryptophan resides, and reveals the conformational changes of proteins during the interaction of ligand-protein (34). Therefore, the measurements of the inner fluorescence spectrum could reveal data on a wide range of molecular activities containing interactions between solvent molecules and fluorophores, rotational diffusion of proteins, distances between sites on biomolecules, conformational changes, and binding interactions (35). As shown in Figure 1, the *λ_*max*_* of SPI is 337–339 nm, which is more than 330 nm, indicating that the Trp in SPI was in a polar microenvironment (36), which was similar to the results observed by Allahdad et al. (6, 7). The fluorescence intensity of SPI decreased after interacting with β-carotene, suggesting that fluorescence quenching occurred, which may be due to the shielding effect of β-carotene binding to the protein chain. As shown in Figures 1A–D, a blue shift of λ_*max*_ from 337–339 nm to 335–336 nm was observed with the increase of β-carotene, indicating that the polarity of the microenvironment decreased and hydrophobic interactions were strengthened, which was in accordance with the result of Deng et al. (19). Soy protein and its subunit underwent aggregation due to the addition of β-carotene.

The fluorescence quenching of a fluorophore induced by a variety of molecular interactions can be dynamic, resulting from collisions between the fluorophore and quencher, or static, resulting from the formation of a ground-state complex between the fluorophore and quencher (37). Kq is one of the determining factors of the quenching mechanisms (6).

As shown in Table 1, the value of Kq is much larger than the diffusion collision constant 2 × 10^10^ L/mol⋅s^–1^, suggesting a static quenching occurred due to the interaction of SPI and β-carotene (38). Additionally, the quenching constant, K_*SV*_, also determined the quenching mechanism. Its values decreased with the increase in temperature, indicating the presence of static quenching (39, 40). Accordingly, it could be confirmed that the SPI–BC complex was formed (41).

**TABLE 1:** Effect of temperature on quenching constants and binding parameters of SPI with β-carotene.

T/K	Stern-volmer			Binding parameters			△H^0^/kJ⋅mol^–1^	△S^0^/J⋅mol^–1^ ⋅K^–1^	△G^0^/J⋅ mol^–1^	R^2^
						
	K_*SV*_/10^5^L⋅mol^–1^	kq/10^13^L⋅mol^–1^ s ^–1^	R^2^	K_*a*_/10^5^ L⋅mol^–1^	n	R^2^				
273	1.07 ± 0.01	1.07 ± 0.01	0.91	0.83 ± 0.01	0.92 ± 0.04	0.90	4,003	32.43	−4,850	0.85
298	0.97 ± 0.02	0.97 ± 0.02	0.98	0.89 ± 0.02	0.97 ± 0.01	0.99			−5,661	
318	0.96 ± 0.03	0.96 ± 0.03	0.98	1.14 ± 0.01	1.05 ± 0.02	0.98			−6,310	
338	0.95 ± 0.07	0.95 ± 0.07	0.96	1.26 ± 0.01	1.10 ± 0.01	0.97			−6,958	

**TABLE 2:** Secondary structure analysis of CDpro based on circular dichroism spectroscopy data.

Temperature	C_β_ (μM/ml)	α-helix	β-sheet	β-turn	Random coil
298K	0	14.6%	35.3%	22.5%	27.6%
	22.35	16.3%	33.1%	25.9%	24.7%
	44.70	16.6%	29.7%	31.2%	22.5%
	67.05	17.3%	28.4%	35.1%	19.2%
	89.40	21.5%	25.6%	36.6%	16.3%
	111.7	27.7%	22.8%	35.0%	14.5%

### Bonding mode analysis

**FIGURE 2:**
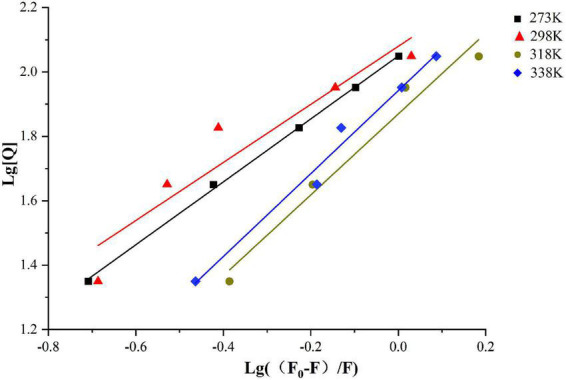
Double logarithmic Stern-Volmer equation liner fit of SPI–BC complex at different thermodynamic temperature (273 K, 298 K, 318 K, and 338 K).

The bonding information including the association constant *K*_*a*_ and the binding stoichiometry were obtained from the double logarithmic regression curve (Figure 2) and provided in Table 1. The *K*_*a*_ value increased with the increase of temperature, suggesting that the complex had a favorable thermostability in the binding system and the interaction was an endothermic reaction. The value was shown to be closed to 1, which revealed that SPI and β-carotene interacted at the molar ratio of 1:1 (42, 43). As described in section “Fluorescence quenching,” different addition ratios of β-carotene caused different changes in the conformation of SPI. Moreover, with the increase of K_*a*_ value, the binding affinity also raised, meaning a more stable bonding occurred, indicating an efficient interaction between SPI and BC that could be an excellent carrier *in vivo* being to take shape, which is consistence with the findings of Chamani et al. (44, 45).

### Thermodynamic property analysis

There are several non-covalent interactive forces between organic ligands and proteins, including van der Waals forces, hydrophobic interaction, hydrogen bonds, and electrostatic interaction (46). According to the results of Ross and Subramanian, if both △H^0^ and △S^0^ are positive, the hydrophobicity is the primary force; if △H^0^ and △S^0^ are negative, Van der Waals force and hydrogen bond are the main interaction (47). To confirm the major interactive force between SPI and β-carotene, free-energy change (△G^0^), enthalpy change (△H^0^), and entropy change (△S^0^) were investigated and listed in Table 1. As shown, both △H^0^ and △S^0^ were positive values indicating that the hydrophobic interaction was the primary driving force. △H^0^ > 0 indicated that the interaction of SPI and β-carotene was an endothermic reaction, which was entropy driven. This was consistent with the previous analysis of K_*a*_, the effective quenching constant for the available fluorophore, whose value trend was similar to the temperature during static quenching, and contrary to K_*SV*_ (40). Additionally, △G^0^ < 0 indicated that the combination of SPI and β-carotene was spontaneous (48), which re-supported the last speculation in section “Bonding mode analysis.”

### Secondary structure analysis

FTIR spectrometry and circular dichroism spectroscopy were conducted to further determine the alteration in protein secondary structure for the qualitative analysis (49). Amide band, consisting of amide I, amide II, and amide III, is the characteristic band for studying protein structures. The amide I band (1,700–1,600 cm ^–1^, C = O tensile vibration) and the amide II band (1,550–1,500 cm ^–1^, N–H bending, and C–N stretching) are most commonly used to characterize the structure of proteins (50).

**FIGURE 3:**
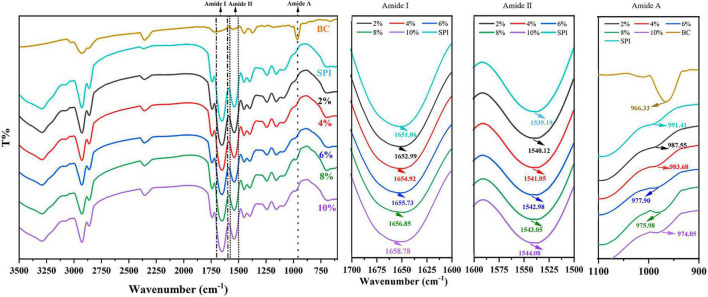
Fourier Transform Infrared Spectroscopy SPI and SPI–BC complex at different weight ratios (BC/SPI: 2%, 4%, 6%, 8%, and 10%).

As shown in Figure 3, SPI showed characteristic peaks around 1,651 cm^–1^ (amide I band, representative of C = O stretching) and 1,539 cm^–1^ (amide II band, representative of N-H bending, and C–N stretching). The amide I band of SPI was weakened after the addition of β-carotene, indicating that β-carotene altered the C = O bond of SPI (51). Meanwhile, the peak position of the amide I band showed a red shift from 1,651 cm^–1^ to 1,653–1,659 cm^–1^, suggesting an increase in α-helix. According to the result of circular dichroism spectroscopy (Table 2), the α-helix increased from 14.6% to 16.3%, 16.6% to 17.3%, 21.5% to 27.7% with the concentration of β-carotene raised from 0 to 22.35 μM/ml, 44.70 to 67.05 μM/ml, 89.40 to 111.7 μM/ml, respectively, which consisted with the fluorescence analysis and the results of Li et al. (52), which manifested the increase of combination between BC and a hydrophilic group of SPI and hydrophobic interaction.

The amide II band (representative of N-H bending), showed a red shift from 1,539 to 1,540–1,544 cm^–1^, implying an increase of β-sheet, which is consistent with the results of the amide I band (53). The outcome of circular dichroism spectroscopy (Table 2) showed the β-sheet decrease from 35.3% to 33.1%, 29.7% to 28.4%, and 25.6% to 22.8% with the concentration of β-carotene raised from 0 to 22.35 μM/ml, 44.70 to 67.05 μM/ml, and 89.40 to 111.7 μM/ml, respectively, verified the result of amide II. Meanwhile, the changes suggested N–H bond of SPI was altered. Alteration of the amide I and amide II bands indicated a possible electrostatic interaction existed between SPI and β-carotene (54, 55). In addition, accompanied by the addition of β-carotene, the intensity of the peak at 964 cm^–1^ raised to 971–994 cm^–1^, which represented the addition of β-carotene altered the conformation of SPI. This phenomenon was regarded as concerned with the decrease of the disorder in protein molecules arrangement (56). The increase of α-helix and the converted changes of random coil certified it.

The secondary structure conversion became more obvious accompanying the addition of β-carotene, convergent with the variation trend of Ka. In other words, the more β-carotene added, the higher the binding affinity and the more powerful interaction force demonstrated by hydrophobic interaction were acquired (57).

### SDS-PAGE analysis

**FIGURE 4:**
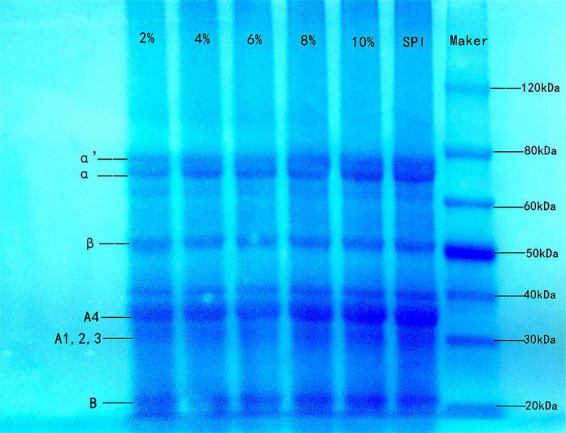
Sodium dodecyl sulfate polyacrylamide gel electrophoresis of SPI and SPI–BC complex at different weight ratios (BC/SPI: 2%, 4%, 6%, 8%, and 10%).

SDS-PAGE is usually used to analyze the molecular weight composition of protein subunits (58). SPI contains two primary globular proteins: 7S (β-conglycinin) and 11S (glycinin). As shown in Figure 4, the SPI–BC complex had intact bands, and no new band was generated compared to native SPI, indicating that there was little impact on SPI molecular weight and no covalent interactions occurred (59). It was speculated that weak intermolecular interactions such as hydrophobic interactions might be the predominated force between SPI and beta-carotene.

### Modular docking

**FIGURE 5:**
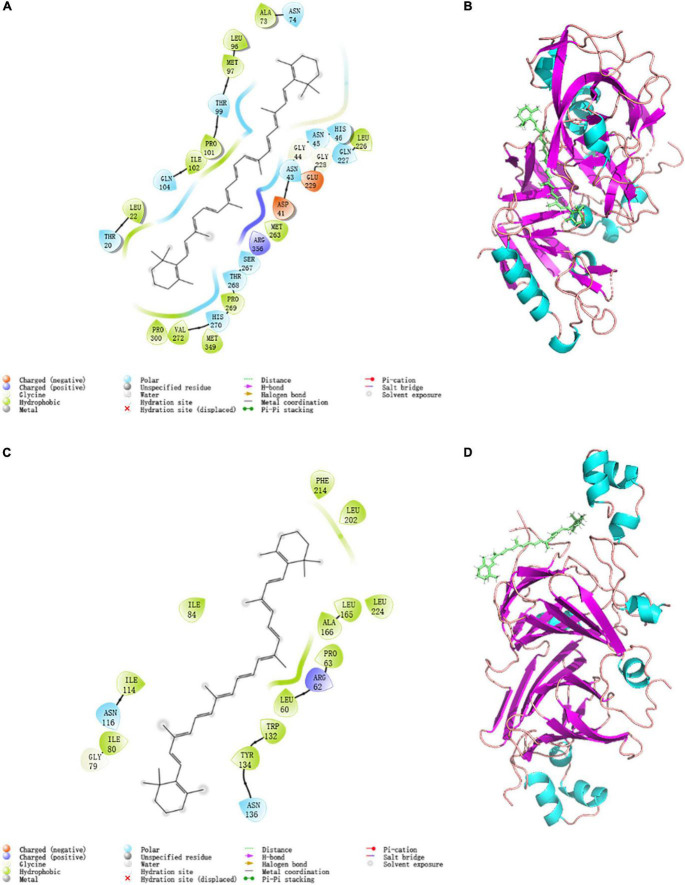
Modular docking 2D and 3D conformational at 298K: **(A)** 7S interactions driving force 2D profile; **(B)** 7S docking result 3D model; **(C)** 11S interactions driving force 2D profile; **(D)** 11S docking result 3D model.

In structural molecular biology, molecular docking is an effective technique, which was widely applied to forecast the primary binding modalities of a ligand with a three-dimensionally known protein (60). Thus the 7S and 11S components of SPI were chosen to dock with β-carotene to evaluate the preferred binding sites and the interaction way. The stereoview of the docking experiments is presented in Figures 5A,B: 7S; Figures 5C,D: 11S.

The topflight binding sites consisting of hydrophobic residues on 7S scoring 1.035 was selected to perform docking. The outcome scored −5.008 with the −4.7903 kJ/mol bonding energy. In the 7S component, hydrophobic interactions dominated the combine and a slight electronic interaction also existed, no H-bond interaction materialized. The specific details are shown in Table 3, it was demonstrated that ALA73, MET97, PRO101, ILE102, LEU22, PRO300, VAL272, MET349, PRO269, MET263, and LEU226 bound with BC through hydrophobic interaction, and the electronic interaction consisted of ARG356, ASP41, and GLU229.

**TABLE 3:** Interactions composition of residues of 7S and 11S.

Protein	Polar	Hydrophobic interaction	Electronic interaction	H-bond
7S	ANS74, THR99, GLN104, THR 20, HIS270, THR263, SER267, ASN43, ASN45, HIS46, GLN27	ALA73, MET97, PRO101, ILE102, LEU22, PRO300, VAL272, MET349, PRO269, MET263, LEU226	ARG356, ASP41, GLU229	None
11S	ASN116, ASN136	ILE80, ILE114, ILE84, PHE241, LEU201. LEU2114, LEU165, ALA166, PRO63, LEU60, TRP132, TYR134	ARG62	None

In the 11S component, a crystal binding site got the highest score of −4.004 with −3.190 kJ/mol bonding energy. The other consequence was similar to 7S and the details were exhibited in Figures 5C,D and Table 3, ILE80, ILE114, ILE84, PHE241, LEU201. LEU2114, LEU165, ALA166, PRO63, LEU60, TRP132, and TYR134 contributed to hydrophobic interaction and only ARG62 made up electronic interaction.

The binding energy was used to assess the binding between the active component and the target, and the binding energy and stability of the ligand–receptor interaction are positively correlated (61). The absolute value of binding energy of 7S was bigger than 11S, meaning that the former had better binding activity and stability, which was consistent with the docking score. Considering the proportion of 7S and 11S is about 3:4 (62), according to the docking result the binding energy was concluded as 4.56 kJ/mol, which was similar to that acquired from fluorescence (4.85 kJ/mol). The fine distinction may cause by the polarity of the solution environment and other components of SPI (6).

Both globulin components bespoke favorable interactions and are mainly influenced by hydrophobic interactions, a potent interaction was formed between SPI and β-carotene, which proved the illations of spectrum analysis.

### DPPH radical scavenging activity

**FIGURE 6:**
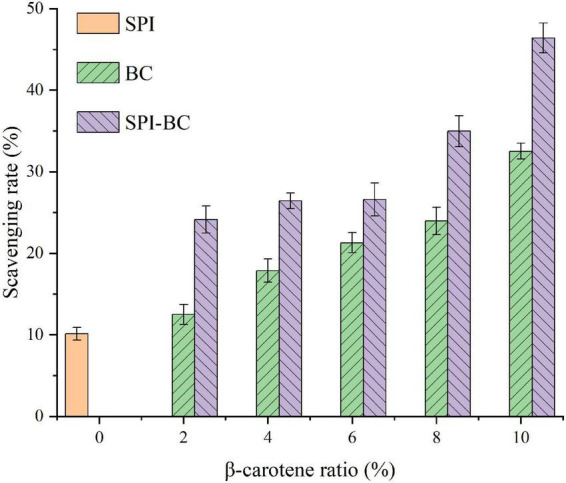
DPPH radical scavenging activity of SPI, free BC and SPI–BC complexes at different weight ratio (BC/SPI: 2%, 4%, 6%, 8%, and 10%).

The DPPH radical scavenging activity was determined to evaluate the changes in the antioxidant capacity of SPI after binding with β-carotene. As shown in Figure 6, the DPPH radical scavenging activity of SPI increased with the increase of BC proportion. Furthermore, the DPPH radical scavenging activity of SPI–BC complex was also higher than free BC, which was consistent with the report of Zhang et al. (21). This may be reasoned by the conformation changes of SPI that the aggregating SPI exposed more free amino groups and the addition of BC improves the affinity of free radicals (59).

### Forming capacity and forming stability

**FIGURE 7:**
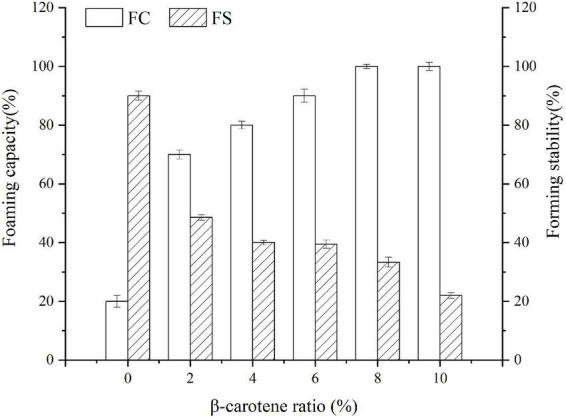
Foaming capacity and forming stability of SPI and SPI–BC complexes at different weight ratio (BC/SPI: 2%, 4%, 6%, 8%, and 10%).

Foaming capacity and forming stability were determined to assess the transformations of protein property. As described in Figure 7, with the weight ratio of BC/SPI raising, the foaming capacity of the SPI–BC complex increased, which was consistent with the study of Deng et al. (31). However, the foaming stability of SPI decreased after binding with BC. This phenomenon can be explained by the fact that the hydrophobic interaction between SPI and BC dominated an action that made the surface hydrophobicity and surface tension raise and form an advanced factor to promote foam formation (63), thus improved the foaming capacity of SPI. Nevertheless, it slowed down the molecules migrating from the aqueous phase to the interface and the surface, thus reducing the foaming stability (64).

### Emulsifying activity index and emulsifying stability index

**FIGURE 8:**
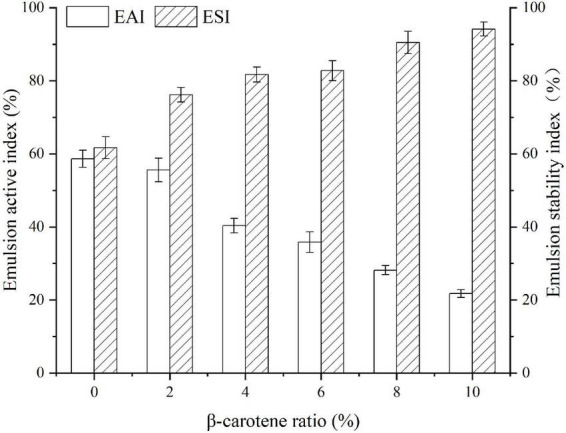
Emulsifying activity index and emulsifying stability index of SPI and SPI–BC complexes at different weight ratio (BC/SPI: 2%, 4%, 6%, 8%, and 10%).

Emulsifying activity index (EAI) is influenced by the number of groups exposed and the molecular migration rate, while emulsifying stability index (ESI) is controlled by the thickness of the interface film and the charge on the surface of the droplet (32, 65). As displayed in Figure 8, with the increase of β-carotene, the EAI of native SPI dropped. That was possible because several structural residues were buried due to the aggregation of SPI after binding with BC, which made it hard to migrate to oil-water interface (32). Whereas, the change in the ESI of the SPI–BC complex was just the opposite on account of the thick interface film intensified by aggregated SPI (66).

## Conclusion

The study found that the combination of SPI and BC was an entropy-driven spontaneous heat-absorbing and can be impacted by changes in ambient variables. The reaction proceeded with being exposed to a polar microenvironment, and the hydrophobic interactions acted as the primary driving force with slight electrostatic interactions. Each SPI molecule has only one binding site for docking with BC, and the stoichiometry is 1:1. After docking with BC, some functional properties of native SPI were enhanced, including DPPH radical scavenging activity, foaming capacity, and emulsifying stability. Infrared spectroscopy, circular dichroism spectroscopy, and fluorescence spectroscopy analysis showed that higher protein aggregation and stronger hydrophobic interactions occurred after the formation of the SPI–BC complex. Although the interaction behavior of SPI and carotene and its effect on protein structure and functional properties were investigated by molecular docking and multispectral analysis, the effect on protein interface properties still needs to be further verified in the process of nutrient delivery. This study is expected to offer some theoretical references for further development of SPI-based functional products rich in beta-carotene in the future.

## Data availability statement

The original contributions presented in this study are included in the article/supplementary material, further inquiries can be directed to the corresponding author/s.

## Author contributions

YZ: funding acquisition, project administration, supervision, writing and editing, and resources. WZ: data curation, writing – original draft, investigation, and validation. ZX and HW: project administration and supervision. RH: investigation, data curation, writing, methodology, and validation. BZ: resources, conceptualization, and supervision. JZ: investigation, validation, and project administration. TL and ZZ: investigation. ZL: funding acquisition, resources, and supervision. All authors contributed to the article and approved the submitted version.

## Footnotes

1
https://pubchem.ncbi.nlm.nih.gov/

2
https://www.rcsb.org/
